# Masking Level Difference: test-retest reliability assessment in normal hearing female university students

**DOI:** 10.1590/2317-1782/20212020207

**Published:** 2022-01-07

**Authors:** Silvana Maria Monte Coelho Frota, Carlos Alberto Leite Filho, Carolina Salomone Bruno, Lanna Borges Carvalho, Natalia Almeida Riegel, Sascha Ariel da Silva Rodrigues de Souza, Fátima Cristina Alves Branco-Barreiro

**Affiliations:** 1 Departamento de Fonoaudiologia, Faculdade de Medicina, Universidade Federal do Rio de Janeiro – UFRJ - Rio de Janeiro (RJ), Brasil.; 2 Programa de Pós-graduação em Ciências da Reabilitação, Departamento de Fisioterapia, Fonoaudiologia e Terapia Ocupacional, Faculdade de Medicina, Universidade de São Paulo – USP - São Paulo (SP), Brasil.; 3 Departamento de Fonoaudiologia, Faculdade de Medicina, Universidade Federal do Rio de Janeiro – UFRJ - Rio de Janeiro (RJ), Brasil.; 4 Departamento de Fonoaudiologia, Universidade Federal de São Paulo – UNIFESP - São Paulo (SP), Brasil.

**Keywords:** Hearing Tests, Reproducibility of Results, Auditory Perception, Auditory Perceptual Disorders, Hearing

## Abstract

**Purpose:**

To verify the test-retest reliability of the Masking Level Difference in normal hearing female university students.

**Methods:**

Prospective descriptive study with 78 young female adults without hearing complaints, submitted to the compact disc version of the Masking Level Difference by Auditec of Saint Louis. The threshold was determined by the difference between signal-to-noise ratios at hearing thresholds found in the antiphasic and homophasic conditions. The test was applied by the same examiner in two moments (test and retest) with an interval of seven to 14 days between them. Inferential statistical analysis included comparison of test and retest situations using Student's t test for paired samples, calculation of the intraclass correlation coefficient and calculation of 95% confidence intervals for signal-to-noise ratios at hearing thresholds found in the antiphasic and homophasic conditions and for masking level difference.

**Results:**

The average signal-to-noise ratio at hearing threshold ​​in the homophasic condition was -12.59 dB and -12.46 dB in the Test and Retest situations, respectively, and -21.54 dB and -21.08 dB in the antiphasic condition. The average value ​​in the final Masking Level Difference result was 8.95 dB in the Test and 8.74 dB in the Retest. Intraclass correlation coefficient values ​​obtained were 0.436, 0.625 and 0.577 for homophasic, antiphasic and Masking Level Difference conditions, respectively.

**Conclusion:**

The Masking Level Difference showed moderate test-retest reliability in normal hearing adults female university students.

## INTRODUCTION

Central Auditory Processing (CAP) is defined as the perceptual processing of auditory information derived from the neurobiological activity in the Central Auditory Nervous System (CANS). The CAP is constituted of mechanisms of auditory discrimination, temporal processing, and binaural processing that originate its hearing skills. Indeed, CAP Disorders is the term used to designate damages in these processes (CAPD)^(1)^.

Performance throughout a behavioral testing battery is an important piece in the CAPD diagnosis puzzle, which includes the Masking Level Difference (MLD) test to assess binaural interaction, that is, the ability of the CANS to process different sound stimuli – complementary or not – introduced on both ears. It consists of listening and synthesis of acoustic information, resulting in a single perceptual event that allows a better hearing performance for benefiting sound source localization and direction, background noise perception, and good performance when associated with competing linguistic message. The results of tests to assess such phenomenon allow inferences on the functional integrity of brainstem – the main structure related to binaural interaction^(1-4)^.

The MLD is based on the masking release phenomenon, described for the first time in 1948 for pure tones^(5)^, which occurs upon the binaural introduction of words or pure tone (generally known “signal”) on both ears, which are sent a narrowband masking noise simultaneously, thus generating auditory competition. The introduction of two stimuli on both ears in homophasic condition, that is, the same sound wave phase, leads to a greater masking noise effect on the signal and consequently higher auditory threshold. Conversely, a weaker noise masker effect on the signal and a lower auditory threshold occur when one of these stimuli is introduced in inverted phase on one ear, characterizing an antiphasic condition. Such improvement characterizes the masking release phenomenon, which can be quantified based on the difference between the thresholds obtained in the monophasic and anti-phase conditions, known as MLD^(6,7)^. This release can contribute to a better understanding on speech situations of competing noise or in the presence of several speakers, since the signal perception is improved when the differences between the binaural tracks of the signal and the masking appear simultaneously^(8)^.

Even though we could not find any other similar studies in adults, tonal MLD proved efficient at distinguishing normal children from those with suspected of CAPD with a sensitivity of 79% and specificity of 88%^(9,10)^. The currently used commercial version of the test was developed in 2003^(11)^, and international studies indicate that a MLD higher than or equal 10 dB is expected for individuals within the normality standards^(3)^. A Brazilian study with the participation of normal-hearing young adults showed an average MLD of 10.83 dB^(12)^.

Tonal MLD is a non-linguistic test that represents an important instrument in a behavioral assessment battery of CAP as it can be applied to individuals with limited linguistic skills or language disorders, in addition to providing simple application and analysis. The main national and international scientific societies in the Audiology field^(13-16)^ recommend the use of a test to assess the auditory skill of binaural interaction and non-linguistic tests in the CAPD diagnosis battery.

The tests used for CAPD diagnosis must provide reliable measures for professionals; therefore, it is fundamental to learn the validity and reliability of the tests contained in the battery to determine the clinical use of these tools^(16)^.

Reliability is among the main quality criteria for an instrument and reflects its capacity to reproduce a result consistently over time. This parameter can be assessed by measuring test-retest reliability, that is, the degree at which similar results are achieved at two distinct moments. Intraclass Correlation Coefficient (ICC) is regarded as the most adequate index to quantify this parameter for reflecting not only its degree of correlation, but also the degree of agreement between situations^(17)^.

Although some studies aimed to establish MLD reliability, important limitations were involved, such as small sample size^(11)^ and samples consisting of children^(18,19)^, while samples composed of adults are recommended for reliability studies due to the maturational stability of the CANS^(20)^. Furthermore, we could not find any national studies addressing the analysis of MLD test-retest reliability in the Brazilian population. Thus, despite its wide clinical use and long existence, MLD is yet to be further explored in studies ranging the stage of reliability verification according to adequate methodological rigor. In this context, our goal was to analyze the MLD test-retest reliability in normal-hearing female university students.

## METHODS

This is a descriptive prospective study carried out at the Outpatient Audiology Clinic of the University Hospital Clementino Fraga Filho at the Federal University of Rio de Janeiro (*Universidade Federal do Rio de Janeiro* (UFRJ)) after being approved by the ethics and research committee of the institution (number 941,370). All participants signed an Informed Consent Form (ICF).

Undergraduate students at the Medical School of UFRJ aged between 20 and 25 years were invited to participate in our study, thus characterizing a convenience sample. The participants were selected by means of sociodemographic and health questionnaire, pure-tone audiometry (250 to 8000 Hz), speech audiometry (Speech Reception Threshold (SRT)), and Binaural integration task in the Dichotic Digit Test (DDT)^(21)^ – here used as CAP screening.

The sample included all students who did not report otological complaints (tinnitus, hearing difficulty, dizziness, ear fullness), history of surgeries, otological changes, acoustic trauma, or neurological alterations. Upon not meeting the following requirements, the subjects were excluded from the sample: audiometry thresholds within the normality standard (≤ 20 dBNA, 250-8000 Hz)^(22)^; SRT compatible with the thresholds found in the three-tone average (500, 1000, and 2000 Hz), and hit ratio above or equal 95% on both ears at the Binaural integration task of DDT^(23)^.

Altogether, 80 subjects were assessed and two were excluded for having presented a hit ratio below 95% in the DDT. Thus, the sample consisted of 78 female young adults aged between 20 and 25 years from undergraduate programs at the Medical School of UFRJ.

The participants were subjected to the commercial version of the tonal MLD test available by the Auditec of Saint Louis, whose recording had an approximate duration of four minutes consisting of the introduction of 33 noise segments at the same phase on both ears along with a pure pulsing tone of 500Hz (signal), in different Signal-to-noise ratios (S/N), in which the signal can be in either of the two following conditions: at the same phase on both ears (homophasic condition – S_o_N_o_) or at inverted phase on one of the ears (antiphasic condition – S_π_N_o_). Furthermore, some of the items in the test were composed only by the noise, thus presenting no signal (No Tone – NT) as control condition. The subject was asked to raise their hand upon hearing the pure tone, thus ignoring the noise masker. By the end of the test application, the hits per condition was quantified and then the MLD was calculated through the equation MLD = S/N in the S_π_N_o_ threshold – S/N in the S_o_N_o_ threshold, in which the threshold corresponding to the number of hits per condition was obtained according to the conversion presented in the test manual^(24)^.

The test was applied in two stages: test and retest, with a time interval from seven to 14 days between, conducted by a single examiner under the same methodological precautions and equipment (Aurical Aud – Software OTOsuite). It is worth emphasizing that the literature recommends a time interval from seven to 14 days for retest^(25-27)^.

The statistical analysis was performed on the SPSS Statistics software, version 25.0 (IBM Corp., Armonk, NY, USA) according to concepts and tools recommended by the literature^(28)^. The descriptive analysis characterized the data collected by calculating the mean, standard deviation, median, and minimum and maximum values. Parametric tests were applied for the inferential analysis since the sample was sufficiently large to allow their direct use due to the Central Limit Theorem^(29)^. The inferential analysis encompassed a comparison of test and retest situations through Student t-test for paired samples, calculation, and interpretation of ICC based on a single measures, absolute agreement, two-way mixed-effects model^(17)^. According to the literature^(17)^, ICCs below 0.5, between 0.5 and 0.75, between 0.76 and 0.9, and above 0.9 were considered to indicate weak, moderate, good, and excellent reliability, respectively. The effect size was measured by calculating the coefficient *d*. Finally, the Confidence Intervals (CI) of 95% were calculated based on bias-corrected and accelerated method for 2000 bootstrap samples.

## RESULTS

We did not find any significant statistical differences between test and retest situations regarding the three MLD conditions, and the comparisons revealed very small effect sizes. Furthermore, the difference between the situations remained ranging the values of -4 and 4 dB, with an average of -0.21 dB and CI of 95% encompassing the value of 0.00 dB, suggesting similar values for both situations (Table 1).

**Table 1:** Descriptive values and comparative analysis of the test-retest situations regarding the MLD parameters

**Parameters**	**Stage**	**Mean**	**SD**	**Median**	**Min.**	**Max.**	***p***	***d***
		**[CI 95%]**		**[CI 95%]**				
S/N S_0_N_0_ (dB)	Test	-12.59	2.60	-12.00	-20.00	-8.00	0.687	0.049
		[-13.13, -12.03]		[-12.00, -12.00]				
	Retest	-12.46	2.66	-12.00	-18.00	-2.00		
		[-13.03, -11.87]		[-12.00, -12.00]				
S/N S_π_N_0_ (dB)	Test	-21.54	2.95	-22.00	-30.00	-14.00	0.118	0.156
		[-22.16, -21.74]		[-22.00, -20.00]				
	Retest	-21.08	3.04	-21.00	-30.00	-14.00		
		[-21.74, -20.44]		[-22.00, -20.00]				
MLD (dB)	Test	8.95	2.34	8.00	4.00	18.00	0.413	0.088
		[8.46, 9.41]		[8.00, 8.00]				
	Retest	8.74	2.44	8.00	2.00	16.00		
		[8.23, 9.26]		[8.00, 8.00]				
MLD – Difference between Test and Retest (dB)	-	-0.21	2.20	0.00	-4.00	4.00	-	-
		[-0.67, 0.26]		[0.00, 0.00]				

Student t-test for paired samples

Caption: MLD: Masking Level Difference; S/N: Signal-to-Noise Ratio; SD: Standard Deviation; Min.: Minimum; Max.: Maximum; CI: Confidence Interval

The mean value of S/N in the auditory threshold at the test-retest stages were -12.59 ± 2.60 dB and -12.46 ± 2.66 dB for homophasic condition, and -21.54 ± 2.95 dB and -21.08 ± 3.04 dB for antiphasic condition, respectively. The mean MLD was 8.95 ± 2.34 dB for the Test, and 8.74 ± 2.44 dB for the Retest.

The ICC to assess reliability through test-retest reached 0.436 for the condition S_o_N_o_, 0.625 for S_π_N_0_, and 0.577 for final MLD (Table 2), indicating weak, moderate, and moderate reliability, respectively^(17)^.

**Table 2:** Analysis of the test-retest reliability regarding MLD

**Parameters**	***n***	**ICC [CI 95%]**	***p***
S/N S_0_N_0_	78	0.436 [0.237. 0.600]	< 0.001^*^
S/N S_π_N_0_	78	0.625 [0.470. 0.743]	< 0.001*
MLD	78	0.577 [0.408. 0.708]	< 0.001*

Caption: MLD: Masking Level Difference; S/N: Signal-to-Noise Ratio; ICC: Intraclass Correlation Coefficient; CI: Confidence Interval;

* statistically significant value at 5% level (p ≤ 0,05)

## DISCUSSION

Although the MLD test has unique characteristics for enabling to assess binaural interaction through non-verbal stimuli, thus contributing to CAPD diagnosis and therapeutic^(2,3,14)^, its reliability, which assures the accuracy of CAPD diagnosis and intervention, is yet to be further studied. Following the trends of other researches^(11,18,19,25)^, this study could establish a comparison between test and retest stages to assess MLD reliability by verifying the occurrence of statistical significant differences between test and retest stages. However, as proposed in more modern statistical approaches^(17-19)^, a more specific assessment of reliability through ICC suggested a moderate degree of test-retest reliability.

Although this study was conducted in female young adults, the few studies on MLD reliability reported in the literature were carried out in children^(18,19)^, except for Wilson et al.^(11)^.

A previous study^(18)^ assessed the MLD test-retest reliability in a group of 24 Norwegian children aged 10 years at an interval of approximately two weeks between test and retest. The authors suggested that the degree of reliability achieved was satisfactory since the ICC value was 0.6 (IC 95%: 0.3-0.8). Even though the age group considered in the study was not the most adequate for studies of test-retest reliability^(20)^, the ICC value obtained was statistically similar to our findings. A possible explanation is the early maturation of the binaural interaction mechanism, as the MLD presents similar results for preschool children and adults^(30)^.

When studying the elaboration and validation of the commercially available version of the MLD test^(11)^, the authors suggested that the instrument provided good reliability based on the absence of significant statistical differences between test and retest stages for 15 college students subjected to the same assessment session. However, in this study, we found a not so optimistic reliability. It is worth highlighting that both studies used different statistical methods to measure test-retest reliability; in addition, the literature suggests that tests for hypothesis of comparing averages of paired samples – as used by Wilson et al.^(11)^ – do not serve such purpose. Furthermore, performing both test and retest in a single session can characterize a very short period to assess reliability due to the influence of factors like memory and learning; therefore, the recommended interval ranges from seven to 14 days^(25-27)^.

An international study^(19)^ assessed the MLD test-retest reliability in 45 English children aged between six and 10 years in a single session and found different results comparing with our study, with a general ICC of 0.36, which can be regarded as indicator of a weak test-retest reliability. However, it is worth highlighting that this latter study included children under seven years old and did not regard the optimum time between the test repetitions, which can characterize a bias. Other factors such as different levels of language development, level of attention, motivation, and understanding regarding the instructions given can justify the unsatisfactory results of the study.

Test-retest reliability establishes the degree at which a certain population is able to maintain the stability of results using a given assessment instrument along time. Thus, it is not a fixed property since many are the factors interfering with the observation of such phenomenon, including sample size, interval between test and retest, studied population, and method of result analysis. Therefore, an instrument may be reliable in certain circumstances, but not in others^(26,27)^.

As to sample size, the literature recommends samples of more than 50 participants to assess test-retest reliability^(25)^, and this study encompasses a sample of 78 normal-hearing adults.

It is known that the interval between test and retest repetitions must be long enough to avoid the effect of memory, but short enough to prevent clinical alterations that could influence the interpretation^(27)^. Therefore, we chose a time interval ranging from seven to 14 days – regarded as adequate to such purpose^(25-27)^.

Regarding the analysis of results, Pearson’s correlation coefficient, t-test for paired samples and Bland-Altman plot are often used for reliability assessment. However, while the former is a simple correlation measure and the latter two only correspond to agreement measures, the ICC indicates both the degrees of correlation and agreement between the measures, thus representing a more desirable reliability measure^(17)^.

We sought to ensure a reliable measurement of the MLD test-retest reliability, and its moderate classification in this study offers some implications to the test interpretation in the context of its clinical practice application. For example, using it in a scenario of pre- and post-auditory training comparison must consider that the test involves a certain degree of instability, consequently, its results must be analyzed carefully and bearing into consideration all other tests in the auditory processing assessment battery.

A potential limitation of our study was associated with the convenience sample including only adults, female university students, despite the prospective descriptive design. The high educational level of such population may have influenced the responses positively. However, it is worth pointing out that previous could not find differences between genders regarding the MLD values^(7,9)^. Furthermore, the lack of research on the Brazilian population narrows the possibilities of comparing studies, leading to the need for further studies to learn the MLD test reliability for male subjects in populations formed by children and elderly, as well as in populations of different socioeconomic or educational levels.

## CONCLUSION

The MLD test showed a moderate degree of test-retest reliability in normal-hearing university students.
