# Supplementary material for: Masking Level Difference: test-retest reliability assessment in normal hearing female university students
Source: Codas. 2022 Jan 7;34(3):e20200207. doi: 10.1590/2317-1782/20212020207 (PMC9851185; doi:10.1590/2317-1782/20212020207)
Supplement: Translation

# Translation: *Masking Level Difference*: avaliação da confiabilidade teste-reteste em estudantes universitárias normo-ouvintes

**Authors:** Silvana Maria Monte Coelho Frota, Carlos Alberto Leite Filho, Carolina Salomone Bruno, Lanna Borges Carvalho, Natalia Almeida Riegel, Sascha Ariel da Silva Rodrigues de Souza, Fátima Cristina Alves Branco-Barreiro

**Affiliations:** 1 Departamento de Fonoaudiologia, Faculdade de Medicina, Universidade Federal do Rio de Janeiro – UFRJ - Rio de Janeiro (RJ), Brasil.; 2 Programa de Pós-graduação em Ciências da Reabilitação, Departamento de Fisioterapia, Fonoaudiologia e Terapia Ocupacional, Faculdade de Medicina, Universidade de São Paulo – USP - São Paulo (SP), Brasil.; 3 Departamento de Fonoaudiologia, Faculdade de Medicina, Universidade Federal do Rio de Janeiro – UFRJ - Rio de Janeiro (RJ), Brasil.; 4 Departamento de Fonoaudiologia, Universidade Federal de São Paulo – UNIFESP - São Paulo (SP), Brasil.

**Keywords:** Descritores Testes Auditivos, Reprodutibilidade dos Testes, Percepção Auditiva, Transtornos da Percepção Auditiva, Audição

## Abstract

**Objetivo:**

Verificar a confiabilidade teste-reteste do *Masking Level Difference* em estudantes universitárias normo-ouvintes.

**Método:**

Estudo prospectivo descritivo com 78 adultos jovens do gênero feminino sem queixas auditivas, submetidas à versão, em *compact disc*, do *Masking Level Difference* da Auditec of Saint Louis. O *Masking Level Difference* foi determinado por meio da diferença entre as relações sinal-ruído nos limiares auditivos encontrados nas condições antifásica e homofásica. O teste foi aplicado pelo mesmo examinador em dois momentos (teste e reteste) com intervalo de sete a 14 dias entre eles. A análise estatística inferencial incluiu comparação das situações teste e reteste por meio do teste t de Student para amostras pareadas, cálculo do coeficiente de correlação intraclasse e dos intervalos de confiança de 95% para as relações sinal/ruído nos limiares auditivos nas condições antifásica e homofásica e para o cálculo do *Masking Level Difference*.

**Resultados:**

A média da relação sinal-ruído no limiar auditivo na condição homofásica foi -12,59 dB e -12,46 dB nas situações teste e reteste, respectivamente, e -21,54 dB e -21,08 dB na condição antifásica. A média do *Masking Level Difference* foi 8,95 dB no teste e 8,74 dB no reteste. Os coeficientes de correlação intraclasse obtidos foram 0,436, 0,625 e 0,577 para as condições homofásica, antifásica e *Masking Level Difference*, respectivamente.

**Conclusão:**

O teste *Masking Level Difference* mostrou grau moderado de confiabilidade teste-reteste em estudantes universitárias normo-ouvintes.

## INTRODUÇÃO

Define-se como processamento auditivo central (PAC) o processamento perceptual da informação auditiva decorrente da atividade neurobiológica do sistema nervoso auditivo central. O PAC é composto pelos mecanismos de discriminação auditiva, processamento temporal e processamento binaural e destes emergem as habilidades auditivas que compõem o PAC. Prejuízos nestes processos são identificados como transtornos do processamento auditivo central (TPAC)^(1)^.

O desempenho na bateria de testes comportamentais é uma peça importante do quebra-cabeça do diagnóstico do TPAC. Dentre os testes comportamentais utilizados para o diagnóstico do PAC está o *Masking Level Difference* (MLD), que avalia a interação binaural, ou seja, a capacidade do sistema nervoso auditivo central de processar estímulos sonoros diferentes, complementares ou não, apresentados nas duas orelhas. Ela consiste na escuta e síntese de informações acústicas, resultando num único evento perceptual que permite melhor desempenho auditivo, pois auxilia na localização e direcionamento da fonte sonora, percepção de ruído de fundo e bom desempenho quando associado à mensagem linguística competitiva. Os resultados dos testes que avaliam este fenômeno permitem inferir sobre a integridade funcional do tronco encefálico, principal estrutura associada à interação binaural^(1-4)^.

O MLD se baseia no fenômeno de liberação do mascaramento, descrito pela primeira vez em 1948 para tons puros^(5)^. Este evento é observado quando da apresentação binaural de palavras ou tom puro (denominados genericamente de “sinal”) nas duas orelhas, que, ao mesmo tempo, recebem um ruído mascarador de banda estreita, gerando competição auditiva. Quando os dois estímulos são apresentados nas duas orelhas em condição homofásica, isto é, mesma fase de onda sonora, observa-se maior efeito de mascaramento do ruído sobre o sinal e, portanto, limiar auditivo mais alto. No entanto, quando um destes estímulos é apresentado com fase invertida em uma das orelhas, caracterizando uma condição antifásica, o efeito mascarador do ruído sobre o sinal é atenuado e observa-se melhora do limiar auditivo. Esta melhora caracteriza o fenômeno de liberação do mascaramento, que pode ser quantificado por meio da diferença entre os limiares obtidos nas condições homofásica e antifásica, denominada MLD^(6,7)^. Essa liberação pode contribuir para uma melhor compreensão da fala em situações de ruído competitivo ou na presença de vários falantes, uma vez que há melhora da percepção do sinal quando as diferenças entre as pistas binaurais do sinal e do mascaramento aparecem simultaneamente no tempo^(8)^.

O MLD tonal se mostrou eficiente para separar crianças normais de crianças com suspeita de transtorno do processamento auditivo central com sensibilidade de 79% e especificidade de 88%^(9,10)^, embora não tenham sido encontrados estudos semelhantes em adultos. A versão comercial do teste utilizada atualmente foi desenvolvida em 2003^(11)^ e estudos internacionais indicam que MLD maior ou igual a 10 dB é esperado em indivíduos dentro dos padrões de normalidade^(3)^. Um estudo brasileiro com adultos jovens normo-ouvintes mostrou média de MLD de 10,83 dB^(12)^.

O MLD tonal é importante para uma bateria comportamental de avaliação do PAC, pois, por ser um teste não-linguístico, pode ser usado em indivíduos com habilidades linguísticas limitadas ou com transtornos de linguagem, além de ser simples e de fácil aplicação e análise. A inclusão de um teste que avalie a habilidade auditiva de interação binaural e de testes não-linguísticos na bateria de diagnóstico do TPAC é recomendada pelas principais sociedades científicas nacionais e internacionais na área da Audiologia^(13-16)^.

Os testes utilizados no diagnóstico do TPAC devem fornecer medidas confiáveis para os profissionais que os utilizam. Portanto, conhecer a validade e a confiabilidade dos testes dessa bateria é crítico para a determinação da utilidade clínica dessas ferramentas^(16)^.

A confiabilidade é um dos principais critérios de qualidade de um instrumento e reflete a sua capacidade de reproduzir um resultado de forma consistente no tempo. Esse parâmetro pode ser avaliado por meio da medida de confiabilidade teste-reteste, ou seja, o grau em que resultados similares são obtidos em dois momentos distintos. O coeficiente de correlação intraclasse (CCI) é considerado o índice mais adequado para quantificação deste parâmetro, uma vez que reflete não só o grau de correlação, mas também o grau de concordância entre as situações^(17)^.

Alguns estudos buscaram estabelecer a confiabilidade do MLD, entretanto apresentaram importantes limitações, como baixo número amostral^(11)^ e amostras compostas por crianças^(18,19)^, sendo que o recomendado para o estudo da confiabilidade é fazê-lo em adultos, devido à estabilidade maturacional do sistema nervoso auditivo central^(20)^. Além disso, não foram encontrados estudos nacionais que tenham analisado a confiabilidade teste-reteste do MLD na população brasileira. Assim, apesar de sua ampla utilização clínica e longo tempo de existência, o MLD ainda carece de estudos que tenham cumprido a etapa de verificação da confiabilidade com rigor metodológico adequado. Deste modo, o objetivo desta pesquisa foi verificar a confiabilidade teste-reteste do MLD em estudantes universitárias normo-ouvintes.

## MÉTODO

O estudo prospectivo descritivo foi realizado no Ambulatório de Audiologia do Hospital Universitário Clementino Fraga Filho da Universidade Federal do Rio de Janeiro (UFRJ) e aprovado pelo comitê de ética e pesquisa da instituição, sob o número 941.370. Todas as participantes do estudo assinaram o Termo de Consentimento Livre e Esclarecido (TCLE).

Foram convidados a participar do estudo estudantes de graduação da Faculdade de Medicina da UFRJ, na faixa etária de 20 a 25 anos, caracterizando uma amostra de conveniência. A seleção das participantes foi feita por meio de questionário sociodemográfico e de saúde, audiometria tonal básica (250 a 8000 Hz), logoaudiometria (Limiar de Reconhecimento de Fala - LRF) e etapa de integração binaural do Teste Dicótico de Dígitos (TDD)^(21)^, utilizado neste estudo como um rastreio do PAC.

Foram incluídas na amostra as estudantes que não referiram queixas otológicas (zumbido, dificuldade auditiva, tontura, plenitude auricular) e sem histórico de cirurgias ou alterações otológicas, trauma acústico ou alterações neurológicas. Foram excluídos da amostra os indivíduos que não cumpriram pelo menos um dos seguintes requisitos: limiares audiométricos dentro do padrão de normalidade (≤ 20 dBNA, 250-8000 Hz)^(22)^; LRF compatível com os limiares encontrados na média tritonal (500, 1000 e 2000 Hz); e índice de acertos maior ou igual a 95% nas duas orelhas na etapa de integração binaural do TDD^(23)^.

Ao todo, 80 indivíduos foram avaliados, sendo que dois foram excluídos por apresentarem índice de acertos menor que 95% no TDD. Assim, a amostra foi composta por 78 adultos jovens do gênero feminino com idades entre 20 e 25 anos, estudantes de cursos de graduação da Faculdade de Medicina da UFRJ.

As participantes foram submetidas ao teste MLD tonal na versão comercializada pela *Auditec of Saint Louis*, cuja gravação tem duração aproximada de quatro minutos e consiste na apresentação de 33 segmentos de ruído na mesma fase nas duas orelhas juntamente com um tom puro pulsátil de 500Hz (sinal), em diferentes relações sinal/ruído (S/R), podendo estar o sinal em duas condições: na mesma fase nas duas orelhas (condição homofásica - S_o_N_o_) ou em fase invertida em uma das orelhas (condição antifásica - S_π_N_o_). Além disso, alguns itens do teste são compostos apenas pelo ruído e sem o sinal (*No Tone* – NT) como condição controle. O indivíduo é orientado a levantar a mão quando ouve o tom puro, ignorando assim o ruído mascarador. Ao final da aplicação do teste, foram quantificados os acertos em cada uma das condições e o cálculo do limiar diferencial de mascaramento foi realizado por meio da equação MLD = S/R no Limiar S_π_N_o_ – S/R no Limiar S_o_N_o_, sendo que o limiar correspondente ao número de acertos para cada condição foi obtido de acordo com a conversão apresentada no manual do teste^(24)^.

O teste foi aplicado em dois momentos: teste e reteste, com um intervalo de tempo de sete a 14 dias entre eles, por um único examinador, sob os mesmos cuidados metodológicos e mesmo equipamento (Aurical Aud – Software OTOsuite). Cabe ressaltar que a literatura recomenda intervalo de tempo de sete a 14 dias para o reteste^(25-27)^.

A análise estatística foi realizada por meio do software SPSS Statistics, versão 25.0 (IBM Corp., Armonk, NY, EUA) e se baseou na utilização de conceitos e ferramentas preconizados pela literatura^(28)^. A análise descritiva caracterizou os dados coletados por meio do cálculo da média, desvio padrão, mediana e valores mínimo e máximo. Para a análise inferencial, foram utilizados testes paramétricos, uma vez que a amostra foi grande o suficiente para permitir a utilização direta destes em virtude do Teorema Central do Limite^(29)^. A análise inferencial incluiu comparação das situações teste e reteste por meio do teste t de Student para amostras pareadas, cálculo e interpretação do coeficiente de correlação intraclasse (CCI) por medidas únicas baseado em um modelo misto de dois fatores do tipo concordância absoluta^(17)^. Conforme recomendado pela literatura^(17)^, CCIs abaixo de 0,5, entre 0,5 e 0,75, entre 0,76 e 0,9 e acima de 0,9 foram considerados, respectivamente, indicativos de confiabilidade fraca, moderada, boa e excelente. O tamanho do efeito foi mensurado por meio do cálculo do coeficiente d. Por fim, para calcular os intervalos de confiança de 95%, utilizou-se o método de viés corrigido e acelerado com base em 2000 amostras bootstrap.

## RESULTADOS

Não foram observadas diferenças estatisticamente significantes entre as situações teste e reteste em relação às três condições do MLD, bem como foram observados tamanhos de efeito muito pequenos para as comparações. Além disso, a diferença entre as duas situações se manteve entre os valores -4 e 4 dB, com média de -0,21 dB e intervalo de confiança de 95% englobando o valor 0,00 dB, sugerindo semelhança dos valores obtidos nas duas situações (Tabela 1).

**Tabela 1:** Valores descritivos e análise comparativa das situações Teste e Reteste em relação aos parâmetros do MLD

**Parâmetros**	**Momento**	**Média**	**DP**	**Mediana**	**Mín.**	**Máx.**	**p**	**d**
		**[IC 95%]**		**[IC 95%]**				
S/R S_0_N_0_ (dB)	Teste	-12,59	2,6	-12,00	-20,00	-8,00	0,687	0,049
		[-13,13, -12,03]		[-12,00, -12,00]				
	Reteste	-12,46	2,66	-12,00	-18,00	-2,00		
		[-13,03, -11,87]		[-12,00, -12,00]				
S/R S_π_N_0_ (dB)	Teste	-21,54	2,95	-22,00	-30,00	-14,00	0,118	0,156
		[-22,16, -21,74]		[-22,00, -20,00]				
	Reteste	-21,08	3,04	-21,00	-30,00	-14,00		
		[-21,74, -20,44]		[-22,00, -20,00]				
MLD (dB)	Teste	8,95	2,34	8,00	4,00	18,00	0,413	0,088
		[8,46, 9,41]		[8,00, 8,00]				
	Reteste	8,74	2,44	8,00	2,00	16,00		
		[8,23, 9,26]		[8,00, 8,00]				
MLD – Diferença entre Teste e Reteste (dB)	-	-0,21	2,2	0,00	-4,00	4,00	-	-
		[-0,67, 0,26]		[0,00, 0,00]				

Teste t de Student para amostras pareadas

Legenda: MLD: Limiar Diferencial de Mascaramento; S/R: Relação Sinal-Ruído; DP: Desvio padrão; Mín.: Mínimo; Máx.: Máximo; IC: Intervalo de confiança

O valor médio da S/R no limiar auditivo dos momentos de Teste e Reteste foram, respectivamente, -12,59 ± 2,60 dB e -12,46 ± 2,66 dB para a condição homofásica e -21,54 ± 2,95 dB e -21,08 ± 3,04 dB para a condição antifásica. A média do MLD foi de 8,95 ± 2,34 dB no Teste e 8,74 ± 2,44 dB no Reteste.

O coeficiente de correlação intraclasse (CCI), que avalia a confiabilidade por meio do teste-reteste, foi de 0,436 para a condição S_o_N_o_, 0,625 para a S_π_N_0_ e de 0,577 para o MLD final (Tabela 2), indicando confiabilidades fraca, moderada e moderada, respectivamente^(17)^.

**Tabela 2:** Análise da confiabilidade teste-reteste do MLD

**Parâmetro**	**n**	**CCI**	**p**
		**[IC 95%]**	
S/R S_0_N_0_	78	0,436	< 0,001*
		[0,237, 0,600]	
S/R S_π_N_0_	78	0,625	< 0,001*
		[0,470, 0,743]	
MLD	78	0,577	< 0,001*
		[0,408, 0,708]	

Legenda: MLD: Limiar Diferencial de Mascaramento; CCI: Coeficiente de correlação intraclasse; IC: Intervalo de confiança;

* valor estatisticamente significante no nível de 5% (p ≤ 0,05)

## DISCUSSÃO

O MLD é um teste com características únicas, uma vez que possibilita a avaliação, por estímulos não-verbais, da interação binaural, contribuindo para o diagnóstico e direcionamento terapêutico do TPAC^(2,3,14)^. Apesar disso, sua confiabilidade, que é determinante na garantia da precisão do diagnóstico e da intervenção no TPAC, ainda foi pouco estudada. No presente estudo, seguindo a tendência de outras pesquisas^(11,18,19,25)^, a comparação dos momentos teste e reteste foi usada para avaliar a confiabilidade do MLD, observando-se ausência de diferenças estatisticamente significantes entre os momentos teste e reteste. Porém, conforme proposto por abordagens estatísticas mais modernas^(17-19)^, a avaliação mais específica da confiabilidade por meio do CCI sugeriu confiabilidade teste-reteste de grau moderado.

Embora o presente estudo tenha sido realizado em adultos jovens do gênero feminino, os poucos estudos sobre a confiabilidade do MLD encontrados na literatura foram realizados com crianças^(18,19)^, com exceção do estudo de Wilson et al.^(11)^.

Um estudo^(18)^ avaliou a confiabilidade teste-reteste do MLD em um grupo de 24 crianças norueguesas de 10 anos com intervalo de aproximadamente duas semanas entre teste e reteste. Os autores sugeriram que o grau de confiabilidade obtido foi satisfatório, pois obtiveram um CCI de 0,6 (IC 95%: 0,3-0,8). Apesar de a faixa etária utilizada pelo estudo não ter sido a mais adequada para estudos de confiabilidade teste-reteste^(20)^, o valor de CCI obtido foi estatisticamente semelhante ao do presente estudo. Uma possível justificativa seria a maturação precoce do mecanismo de interação binaural, sendo que crianças pré-escolares já apresentam resultados semelhantes aos de adultos no MLD^(30)^.

No estudo de desenvolvimento e validação da versão do teste MLD disponível comercialmente^(11)^, os autores sugeriram que o instrumento tem boa confiabilidade ao não encontrarem diferenças estatisticamente significantes entre os momentos teste e reteste para 15 universitários numa mesma sessão de avaliação. No presente estudo, a confiabilidade encontrada foi menos otimista. É válido salientar que as duas pesquisas utilizaram métodos estatísticos diferentes para mensuração da confiabilidade teste-reteste e que a literatura sugere que testes de hipótese para comparação de médias de amostras pareadas, como o utilizado por Wilson et al.^(11)^, não são suficientes para este fim. Além disso, a realização de teste e reteste numa mesma sessão pode caracterizar um período muito curto para avaliar a confiabilidade devido à influência de fatores como memória e aprendizagem, sendo recomendado o intervalo de 7 a 14 dias^(25-27)^.

Ao avaliar a confiabilidade teste-reteste do MLD em 45 crianças inglesas de seis a 10 anos numa mesma sessão, um estudo internacional^(19)^ obteve resultados discrepantes aos do presente estudo ao observar CCI geral de 0,36, que pode ser considerado indicativo de uma confiabilidade teste-reteste fraca. Porém, vale ressaltar que esse último estudo foi realizado com crianças abaixo de sete anos e sem respeitar o período de tempo ideal entre a repetição das administrações do teste, o que pode ser considerado um viés. Fatores outros como diferentes níveis de desenvolvimento de linguagem, nível de atenção, motivação e entendimento das instruções dadas para a realização do teste podem ajudar a justificar os resultados insatisfatórios desse estudo.

A confiabilidade teste-reteste estabelece o grau no qual uma determinada população é capaz de manter a estabilidade de seus resultados em um determinado instrumento de avaliação ao longo do tempo. Assim, não se trata de uma propriedade fixa, de tal maneira que diversos fatores podem interferir na observação deste fenômeno, incluindo tamanho amostral, tempo entre teste e reteste, população estudada e método de análise dos resultados. Dessa forma, um instrumento pode ser confiável em determinadas circunstâncias e em outras, não^(26,27)^.

Quanto ao tamanho amostral, este estudo contou com uma amostra de 78 adultos normo-ouvintes, sendo que a literatura recomenda amostras superiores a 50 participantes para avaliação da confiabilidade teste-reteste^(25)^.

Em relação ao tempo entre teste e reteste, sabe-se que o período entre a repetição das administrações deve ser longo o suficiente para impedir o efeito da memória, mas curto o suficiente para garantir que não ocorram alterações clínicas, influenciando sua interpretação^(27)^. Deste modo, escolheu-se um intervalo de tempo de sete a 14 dias, considerado adequado para esse fim^(25-27)^.

Quanto à análise dos resultados, o coeficiente de correlação de Pearson, o teste t para amostras pareadas e o gráfico de Bland-Altman são muitas vezes utilizados para avaliar a confiabilidade. No entanto, enquanto o primeiro se trata apenas de uma medida de correlação e os dois últimos correspondem apenas a medidas de concordância, o CCI reflete tanto o grau de correlação quanto a concordância entre as medidas, representando uma medida de confiabilidade mais desejável^(17)^.

Buscou-se, portanto, garantir a mensuração fidedigna da confiabilidade teste-reteste do MLD. O fato de esta ter sido classificada como moderada traz algumas implicações na interpretação do teste no contexto da sua utilização na prática clínica. Por exemplo, seu uso em uma situação de comparação pré e pós-treinamento auditivo deve levar em consideração que o teste possui certo grau de instabilidade e, portanto, seus resultados devem ser analisados com cautela e levando em consideração os demais testes da bateria de avaliação do processamento auditivo.

Uma possível limitação deste estudo se deve ao fato que, mesmo sendo um estudo com delineamento prospectivo descritivo, a amostra da pesquisa foi de conveniência, formada somente por adultos, estudantes universitários e do gênero feminino. Portanto, nessa população o nível de escolaridade é alto, o que pode ter influenciado positivamente nas respostas. No entanto, é válido notar que estudos anteriores não encontraram diferenças entre os gêneros em relação aos valores do MLD^(7,9)^. Além disso, a escassez de pesquisas na população brasileira reduz as possibilidades de comparações entre estudos. Assim, observa-se a necessidade de outros estudos para conhecer a confiabilidade do teste MLD no gênero masculino, em populações formada por crianças e idosos e em populações de diferentes níveis socioeconômicos ou de escolaridade.

## CONCLUSÃO

O teste *Masking Level Difference* (MLD) mostrou grau moderado de confiabilidade teste-reteste em estudantes universitárias normo-ouvintes.
